# miR-324-5p and miR-30c-2-3p Alter Renal Mineralocorticoid Receptor Signaling under Hypertonicity

**DOI:** 10.3390/cells11091377

**Published:** 2022-04-19

**Authors:** Thi An Vu, Ingrid Lema, Imene Hani, Lydie Cheval, Laura Atger-Lallier, Vilayvane Souvannarath, Julie Perrot, Mélanie Souvanheuane, Yannick Marie, Sylvie Fabrega, Anne Blanchard, Jérôme Bouligand, Peter Kamenickỷ, Gilles Crambert, Laetitia Martinerie, Marc Lombès, Say Viengchareun

**Affiliations:** 1 Physiologie et Physiopathologie Endocriniennes, Université Paris-Saclay, Inserm, 94276 Le Kremlin-Bicêtre, France; thi-an.vu@universite-paris-saclay.fr (T.A.V.); lemaingrid@hotmail.fr (I.L.); imene.hani@universite-paris-saclay.fr (I.H.); laura.atger@gmail.com (L.A.-L.); vilayvane.souvannarath@outlook.com (V.S.); julie.perrot@inserm.fr (J.P.); melanie@souvanheuane.com (M.S.); jerome.bouligand@aphp.fr (J.B.); peter.kamenicky@aphp.fr (P.K.); laetitia.martinerie@aphp.fr (L.M.); marc.lombes@universite-paris-saclay.fr (M.L.); 2 Centre de Recherche des Cordeliers, Inserm, Sorbonne Université, Université Paris Cité, 75006 Paris, France; lydie.cheval@crc.jussieu.fr (L.C.); gilles.crambert@crc.jussieu.fr (G.C.); 3 Plateforme de Genotypage Séquençage (iGenSeq), Institut du Cerveau et de la Moelle Epinière, Hôpital Sapêtrière, 75013 Paris, France; yannick.marie@icm-institute.org; 4 Plateforme Vecteurs Viraux et Transfert de Gènes, Structure Federative de Recherche Necker, UMS 24, UMS 3633, Faculté de Santé, Université Paris Cité, 75015 Paris, France; sylvie.fabrega@inserm.fr; 5 Inserm, Centre d’Investigations Cliniques 9201, 75015 Paris, France; anne.blanchard@aphp.fr; 6 Assistance Publique-Hôpitaux de Paris, Hôpital Bicêtre, Service de Génétique Moléculaire, Pharmacogénétique et Hormonologie, 94275 Le Kremlin-Bicêtre, France; 7 Assistance Publique-Hopitaux de Paris, Hôpital Bicêtre, Service d’Endocrinologie et des Maladies de la Reproduction, 94275 Le Kremlin-Bicêtre, France; 8 Assistance-Publique Hôpitaux de Paris, Hôpital Robert Debré, Service d’Endocrinologie Pédiatrique, Université Paris Cité, 75019 Paris, France

**Keywords:** microRNAs, mineralocorticoid receptor, aldosterone, hypertonicity, post-transcriptional regulation, sodium reabsorption

## Abstract

The Mineralocorticoid Receptor (MR) mediates the sodium-retaining action of aldosterone in the distal nephron, but mechanisms regulating MR expression are still poorly understood. We previously showed that RNA Binding Proteins (RBPs) regulate MR expression at the post-transcriptional level in response to variations of extracellular tonicity. Herein, we highlight a novel regulatory mechanism involving the recruitment of microRNAs (miRNAs) under hypertonicity. RT-qPCR validated miRNAs candidates identified by high throughput screening approaches and transfection of a luciferase reporter construct together with miRNAs Mimics or Inhibitors demonstrated their functional interaction with target transcripts. Overexpression strategies using Mimics or lentivirus revealed the impact on MR expression and signaling in renal KC3AC1 cells. miR-324-5p and miR-30c-2-3p expression are increased under hypertonicity in KC3AC1 cells. These miRNAs directly affect *Nr3c2* (MR) transcript stability, act with Tis11b to destabilize MR transcript but also repress *Elavl1* (HuR) transcript, which enhances MR expression and signaling. Overexpression of miR-324-5p and miR-30c-2-3p alter MR expression and signaling in KC3AC1 cells with blunted responses in terms of aldosterone-regulated genes expression. We also confirm that their expression is increased by hypertonicity in vivo in the kidneys of mice treated with furosemide. These findings may have major implications for the pathogenesis of renal dysfunctions, sodium retention, and mineralocorticoid resistance.

## 1. Introduction

The Mineralocorticoid Receptor (MR), encoded by the *NR3C2* gene, belongs to the nuclear receptor superfamily and mediates aldosterone action by stimulating transcription of target genes involved in the control of sodium reabsorption in tight epithelia [1], such as the α subunit of the Epithelial Na^+^ Channel [2,3,4], the Serum and glucocorticoid-regulated kinase 1 (*Sgk 1*) [5,6], or the Glucocorticoid-induced leucine zipper transcription factor (*Gilz*) [7]. Thus, MR controls salt and water balance and participates in regulating blood pressure. This MR signaling is not functional in newborns due to low renal MR expression at birth, which accounts for the physiologic partial aldosterone resistance of human newborns, reminiscent of the MR haploinsufficiency reported in autosomal dominant pseudohypoaldosteronism [8]. Moreover, many studies showed that the MR plays a pivotal role in the pathogenesis of several diseases, including heart failure, hypertension [9], and nephropathies [10]. However, mechanisms controlling renal MR expression remain poorly understood. At the transcriptional level, the human *NR3C2* gene is driven by two alternative promoters, the proximal P1 promoter, which is transcriptionally active in all MR target tissues, and the distal P2 promoter, which is weaker and transcriptionally active in the central nervous system [11,12]. Beyond these transcriptional regulatory mechanisms, MR activity and signaling are also modulated by post-translational modifications such as phosphorylation, SUMOylation, ubiquitylation, and acetylation [1,13]. In the distal nephron, MR is mainly expressed in the cortex, where the tubular lumen is rather hypotonic, while its expression is low in the medulla [14,15], which is hypertonic. Previously, we have demonstrated that variations of extracellular tonicity, prevailing in nephron segments, modulate MR expression through post-transcriptional mechanisms involving the recruitment of RNA Binding Proteins (RBPs) [16]. Indeed, we showed that hypertonicity compromises MR signaling by recruiting Tis11b (Tetradecanoyl phorbol acetate inducible sequence 11b), which degrades MR mRNA [17], while HuR (Human antigen R) favors MR mRNA stability [18] and edits MR mRNA [19], thus enhancing MR signaling under hypotonicity. In this context, we could not exclude another working hypothesis involving the recruitment of microRNAs (miRNAs), another class of post-transcriptional regulators. These endogenous short non-coding RNAs [20,21], which negatively regulate gene expression mainly by targeting the 3′-Untranslated Region (3′-UTR) of target transcripts, are involved in various biological processes [22,23,24]. The miRNAs-target gene interaction constitutes a complex network. Indeed, overexpression of a particular miRNA affects the expression of hundreds of target transcripts [25,26]. Conversely, one specific transcript is generally regulated by several miRNAs [27]. Furthermore, it was shown that miRNAs can be secreted in plasma or urine and might thus constitute promising biomarkers, as well as potential novel therapeutic targets for renal dysfunction [28,29]. Indeed, accumulating evidence now suggests that miRNAs exert key roles not only in development but also in renal diseases [30,31,32], especially those affecting MR signaling [33]. For instance, putative binding sites for miRNAs were identified in the 3′-UTR of transcripts related to the renin-angiotensin-aldosterone system [34]. miR-34c-5p was shown to play an important role in aldosterone-induced fibrosis in the kidney [35]. miRNAs were also shown to impact ionic transport in response to aldosterone [36,37,38,39,40] or MR expression in other pathophysiological situations [34,41,42,43,44]. However, no study reported the role of miRNAs on MR expression in response to tonicity. We have identified miR-324-5p and miR-30c-2-3p, the expression of which is upregulated by hypertonicity in murine principal KC3AC1 cells. Herein, we describe how they functionally interact with MR transcript, in cooperation with RBPs, and subsequently affect its expression and MR signaling. In vivo, we have further demonstrated that their expression is significantly increased in the kidneys of mice treated with furosemide, a diuretic known to increase the relative luminal tonicity in the distal nephron.

## 2. Materials and Methods

### 2.1. Cell Culture

KC3AC1 cells were obtained by a targeted oncogenesis strategy in a transgenic mouse model in which expression of the T Antigen of SV40 was placed under the control of the proximal promoter of the human *NR3C2* gene. These principal cells, which endogenously express MR, were isolated from the cortical collecting ducts [16]. Cells were cultured under isotonic medium (300 mOsM/kg) with DMEM/HAM’S F12 supplemented with 5% dextran charcoal-coated calf serum (DCC), 2 mM glutamine, 20 mM HEPES, pH 7.4, 100 U/mL penicillin, and 100 µg/mL streptomycin (Life Technologies, Villebon-sur-Yvette, France), 5 µg/mL Insulin (Sigma, Saint-Quentin-Fallavier, France), 5 µg/mL transferrin (Sigma, Saint-Quentin-Fallavier, France), 50 nM sodium selenite (Sigma, Saint-Quentin-Fallavier, France), 50 nM dexamethasone (Sigma, Saint-Quentin-Fallavier, France), 2 nM triiodothyronine T3 (Sigma, Saint-Quentin-Fallavier, France), 10 ng/mL epithelial growth factor (EGF) (PeproTech, Neuilly-sur-Seine, France). Hypertonic conditions (500 mOsM/kg) were achieved by adding 0.2 M raffinose (Sigma, Saint-Quentin-Fallavier, France). Minimal medium lacking dexamethasone, EGF, and DCC was used to study aldosterone action (Acros Organics, Villebon-sur-Yvette, France). HEK 293T cells were cultured in DMEM supplemented with 10% fetal calf serum (Biowest, Courtaboeuf, France), 2 mM glutamine (Life Technologies, Villebon-sur-Yvette, France), 20 mM HEPES, pH 7.4 (Life Technologies, Villebon-sur-Yvette, France), 100 U/mL penicillin (Life Technologies, Villebon-sur-Yvette, France), and 100 µg/mL streptomycin (Life Technologies, Villebon-sur-Yvette, France).

### 2.2. Total RNAs Isolation

RNAs, isolated from cultured cells or from mouse kidneys, were extracted with NucleoSpin^®^ miRNA kit (Macherey Nagel, Hoerdt, France) according to the manufacturer’s protocol. This kit facilitates the isolation of both miRNAs and large RNAs from each sample for use in RT-qPCR, Taqman Low-Density Array analysis, and miRNAs-sequencing.

### 2.3. Taqman Low Density Array (TLDA)

TLDA allows testing of a large subset of miRNAs by RT-qPCR in a single assay. First, 600 ng of miRNAs from isotonic or hypertonic medium-incubated KC3AC1 cells were subjected to reverse transcription (RT) using the TaqMan^®^ miRNA reverse transcription kit and Megaplex^TM^ RT primers Pool Set v2.0 (Pool A and B) according to the manufacturer’s protocol (Applied Biosystems, Villebon-sur-Yvette, France). An amplification step was then performed in the Service of Molecular Genetics, Pharmacogenetics, and Hormonology of Bicetre Hospital (France). RT products were diluted with RNAse-free water, combined with TaqMan^®^ 2X Universal PCR Master Mix, No AmpErase UNG then loaded into four TaqMan^®^ rodent microRNA array v2.0 (Applied Biosystems, Villebon-sur-Yvette, France: one Array A and one Array B for isotonic or hypertonic condition), which are 384-well microfluidic cards embedded TaqMan probes in each well for different mature rodent miRNAs enabling accurate quantification of 585 miRNAs for mice for each condition. snoRNA 202 transcript was used for normalization. Quantitative PCR was performed according to the manufacturer’s instructions and ran on a 7900 HT Fast Real-Time PCR system with 384-Well Block Module (Applied Biosystems, Villebon-sur-Yvette, France). Results were analyzed on SDS 2.3 software (Applied Biosystems, Villebon-sur-Yvette, France) and expressed as 2^−^^ΔΔCt^ values with ΔCt = Ct_miRNA_ − Ct_snoRNA 202_.

### 2.4. miRNAs-Sequencing (miRNAs-Seq)

miRNAs-Seq was performed to profile different miRNAs under isotonic or hypertonic medium-incubated KC3AC1 cells. An amount of 200 ng total RNAs (containing miRNAs) per sample (*n* = 4 per condition) was used to conduct miRNAs library preparation and sequencing at iGenSeq Platform (Institut du Cerveau et de la Moelle Epinière, Paris, France). The concentration and size distribution of all samples were determined by 4200 TapeStation (Agilent, Les Ulis, France). Library preparation for sequencing was performed on the RNA fragment under 200 bases by using the QIAseq^®^ miRNA Library kit according to the manufacturer’s protocols (Qiagen, Villebon-sur-Yvette, France). Briefly, a pre-adenylated DNA adapter was ligated to the 3′-end of all mature miRNAs; an RNA adapter was ligated to the 5′-end of mature miRNAs. Ligated miRNAs were reverse transcribed to cDNA using a reverse transcription (RT) primer with integrated Unique Molecular Indices (UMIs), which are molecular tags used to detect and quantify unique mRNA transcripts. After reverse transcription, a cleanup of the cDNA was performed using a streamlined magnetic bead-based method before and after library amplification. Next-generation Sequencing was performed in a single-end mode, 1 × 75 bp, on the NextSeq 500 Illumina System platform with approximately 32 million reads per sample, following the manufacturer’s protocols. Reads quality was assessed using FastQC on the FASTQ file. Alignment was performed against the mouse genome (GRCh38/mm10). Raw miRNA counts and differential expression were analyzed using R/Bioconductor package DESeq2. An adjusted *p*-value < 0.05 with Benjamini & Hochberg False Discovery Rate method was considered statistically significant.

### 2.5. miRNAs RT-qPCR

For miRNAs RT-qPCR, a multiplex reverse transcription protocol was performed to reverse transcribe simultaneous multiple miRNAs (12 miRNAs maximum) into cDNA from 200 ng of miRNAs using the TaqMan^®^ microRNA Reverse Transcription kit (Applied Biosystems, Villebon-sur-Yvette, France) and diluted (1:100 in 1X TE) specific stem-loop primers for corresponding miRNAs (Applied Biosystems, Villebon-sur-Yvette, France). RT products were used for quantification of individual miRNAs that were run on a QuantStudio 6 Flex (Applied Biosystems, Villebon-sur-Yvette, France) using specific miRNAs TaqMan probes (Life Technologies, Villebon-sur-Yvette, France) and TaqMan Universal PCR Master Mix, No AmpErase UNG, 2X (Life Technologies, Villebon-sur-Yvette, France). A list of specific miRNA stem-loop primers and TaqMan probes is presented in Supplementary Table S1. The specificity of each miRNA TaqMan probe was confirmed by cloning each amplicon into the pGEMT-easy plasmid (Promega, Charbonnière, France) and sequencing. The efficiency of qPCR was controlled before analysis. Relative expression in each miRNA was normalized to the housekeeping miRNA (mmu-miR-16 or snoRNA 202), where control condition values were arbitrarily set at 100%.

### 2.6. mRNAs RT-qPCR

For mRNAs RT-qPCR, after DNAse I treatment (Biolabs, Évry-Courcouronnes, France), 1 µg of total RNAs was reverse transcribed using the High-Capacity cDNA reverse transcription kit (Life Technologies, Villebon-sur-Yvette, France). Samples were analyzed by RT-qPCR using the Power SYBR^®^ Green PCR Master Mix (Life Technologies, Villebon-sur-Yvette, France) with the primers indicated in Supplementary Table S2A and run on QS6 Real-Time PCR System (Life Technologies, Villebon-sur-Yvette, France). The specificity of each primer set was confirmed by cloning each amplicon into the pGEMT-easy plasmid (Promega, Charbonnière, France) and sequencing. qPCR Efficiency was at least 95% in all experiments. Relative expression in each sample was calculated as a ratio (amol of specific gene/fmol of 18S or amol of 36b4), where the control condition values are arbitrarily set at 100%.

### 2.7. Plasmid Constructs

Wild type murine *Elavl1* (HuR) 3′-UTR (mHuR 3′-UTR) was amplified by PCR from cDNA using specific primers presented in Supplementary Table S2B, in which *SpeI* and *HindIII* restriction sites were introduced to facilitate cloning into the pMIR-Report^TM^ plasmid (Life Technologies, Villebon-sur-Yvette, France). pMIR-mMR-3′-UTR plasmid and Tis11b-encoding pTarget vector were generated as previously described [18]. All plasmids were sequenced to verify nucleotide identity at the Eurofins Genomics platform (Cologne, Germany).

### 2.8. Luciferase Assays

To study the functional interaction between miRNAs and *Nr3c2* (MR) or *Elavl1* (HuR) 3′-UTR, HEK 293T cells were transfected, using Lipofectamine 2000, with the luciferase constructs (40 ng/well of 96-well plates) with either increasing concentrations of Mimics or negative control Mimics (CTR Mimics) or with a maximal effective concentration of Inhibitors or negative control Inhibitors (CTR Inhibitors) (Life Technologies, Villebon-sur-Yvette, France, see sequences in Supplementary Table S3. To evaluate the cooperativity between miRNAs and the Tis11b RNA-Binding Protein on *Nr3c2* (MR) and *Elavl1* (HuR) 3′-UTR, the Tis11b-encoding pTarget vector (10 ng/well of 96-well plates) was cotransfected into HEK 293T together with pMIR-mMR-3′-UTR (40 ng) or pMIR-mHuR-3′-UTR (40 ng) with or without Mimics or control Mimics. Cell lysates were collected 24 h post-transfection to measure luciferase activities. Luciferase activities were normalized to β-galactosidase activities as previously described [17].

### 2.9. Modulation of miR-30c-2-3p Expression

KC3AC1 cells, seeded in 12 well-plates (300,000 cells/well), were transiently transfected on day 2 of cell culture, using Lipofectamine RNAiMax reagent (Life Technologies, Villebon-sur-Yvette, France), with 10 nM 30c-2-3p Mimics or CTR Mimic to overexpress miR-30c-2-3p or with 10 nM 30c-2-3p Inhibitors or CTR Inhibitor to repress miR-30c-2-3p expression. To study the functional consequences of miR-30c-2-3p overexpression on MR signaling, renal cells were deprived for 24 h in a minimal medium and then treated for 1 h with 10 nM aldosterone. Total RNAs and protein extracts were processed for RT-qPCR and Western Blot analysis after 18 h and 48 h, respectively.

### 2.10. Establishment of KC3AC1 Clones Stably Transduced with Lentivirus Expressing Inducible miR-324-5p

Production of HIV-1 U3-SIN lentivirus particles was carried out at the Viral Vector and Gene Transfert Platform (Structure Federative de Recherche Necker, UMS 24, UMS 3633, Faculté de Santé, Université Paris Cité, France) by cotransfection into HEK 293T cells of the miR-324-5p inducible lentiviral plasmid (ShMIMIC Inducible miR-324-5p, #GSH11929-224640456, Dharmacon, Cambridge, United Kingdom) or the control inducible lentiviral plasmid (SMARTvector Inducible Non-targeting mCMV-TurboRFP, #VSC1165, Dharmacon, Cambridge, United Kingdom) with the vesicular stomatitis G protein (VSV-G) envelope, and other packaging plasmids using the standard calcium phosphate transfection method. After production, functional lentiviral titers were determined in hHTC 116 cells by puromycin selection or Red Fluorescent protein Fluorescence-Activated Cell Sorting analysis 48 h after induction by doxycycline (Sigma, Saint-Quentin-Fallavier, France) at 1 µg/mL. The harvested supernatant containing ShMIMIC miR-324-5p or scrambled lentiviral particles was transduced into renal KC3AC1 cells at a multiplicity of infection (MOI) of 20. The selection of transduced cells was performed by adding 1.5 µg/mL of puromycin (InvivoGen, Toulouse, France) 48 h after transduction. After 5 days of selection, cells were subjected to serial dilutions in 96-well plates and maintained in culture for up to 15 days until the expansion of isolated clones. After 48 h incubation, doxycycline (1 µg/mL), KC3AC1 clones overexpressing miR-324-5p could be visualized thanks to the observation of the Red Fluorescent Protein (excitation at 553 nm/emission at 574 nm) on BX73 optical microscope (Olympus). Finally, miRNAs RT-qPCR analyses allowed selection of the clone Sm-A1, which stably overexpresses scrambled control miRNAs, and the clone Sh-H8, which, on the opposite, stably overexpresses the miR-324-5p. Sm-A1 and Sh-H8 clones were cultured in the same medium as the parental KC3AC1 cells with puromycin (1 µg/mL). To analyze the impact of miR-324-5p overexpression on MR signaling, Sh-A1 and Sh-H8 clones cultured on filters onto 12 well-plates (1.2 × 10^6^ cells/filter) were deprived in minimal medium lacking dexamethasone, EGF, DCC concurrently subjected to doxycycline (1 µg/mL) induction for 48 h then stimulated with 10 nM aldosterone for 1 h. Total RNAs were thereafter extracted then relative MR target gene expression was analyzed by RT-qPCR.

### 2.11. Western Blot Analyses

Forty micrograms of extracted proteins were subjected to SDS-PAGE and processed for detection of MR protein together with α-tubulin protein for loading normalization. The signal fluorescence intensity was determined with an Odyssey^®^ (Li-Cor, Homburg, Germany). Antibody sources and dilutions are given in Supplementary Table S4.

### 2.12. Investigations in Mice

Mice received an intraperitoneal injection of furosemide (40 mg/kg, Renaudin, Itxassou, France) for 4 h. Animals (*n* = 5–6 per group) were euthanized, and kidneys were collected and snap-frozen in liquid nitrogen for subsequent analyses. Mice were bred according to the Guide for the Care and Use of Laboratory Animals published by the National Institutes of Health (NIH Publication No. 85-23, revised 1996). The animal facility was granted approval (no. C94–043-12) by the Ministère de l’Agriculture, France. All procedures were approved by the local ethic committee CAPSud (N°2012-021).

### 2.13. Statistical Analyses

Data are means ± SEMs. *t*-test with Welch’s correction or One-way ANOVA followed by Bonferroni’s post-hoc test was used when appropriate to determine significant differences (GraphPad 6.0 Prism software, San Diego, CA, United States). A *p*-value below 0.05 was considered as statistically significant (* or ^§^ *p* < 0.05; ** or ^§§^ *p* < 0.01; *** or ^§§§^ *p* < 0.001; **** or ^§§§§^ *p* < 0.0001).

## 3. Results

### 3.1. Hypertonicity Induces miRNAs Expression

**Figure 1:**
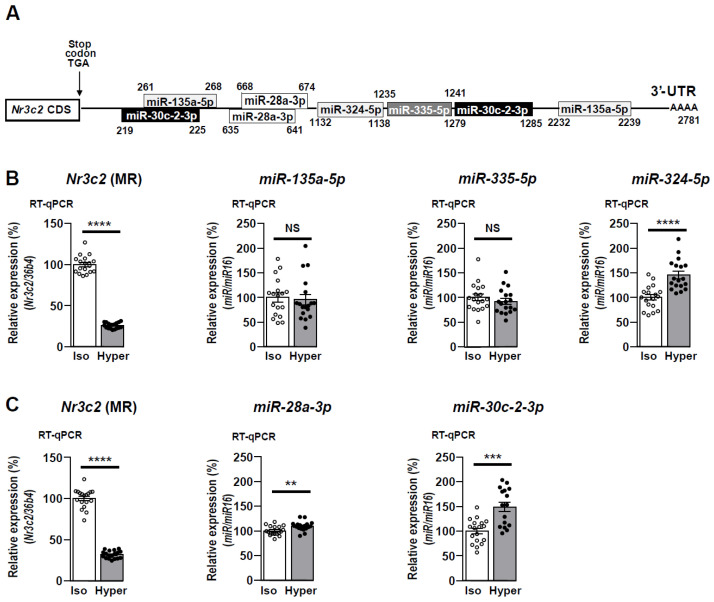
Schematic representation of *Nr3c2* (MR) 3′-UTR, expression of *Nr3c2* (MR) and miRNA candidates in renal KC3AC1 cells under hypertonicity. (**A**) Location of predicted binding sites for miRNAs in the murine *Nr3c2* (MR) 3′-UTR, 2.78 kbp, positioned after the stop codon TGA, arbitrarily set at +1. (**B**,**C**) RT-qPCR analyses of renal MR and expression of miRNA candidates from TLDA (**B**) and miRNAs-seq (**C**) approaches. KC3AC1 cells were grown for 7 days in complete medium then cells were exposed to isotonicity (Iso) or hypertonicity (Hyper) for 6 h. *Nr3c2* (MR) transcript and miRNA levels under hypertonicity are expressed as a percentage of *Nr3c2* (MR) mRNA or miRNA levels under isotonicity (arbitrarily set at 100%). Data are means ± SEMs from three independent experiments performed in six replicates (*n* = 18); isotonic condition (open circle, o), hypertonic condition (black circle, •). NS = not significant, ** p < 0.01, *** p < 0.001, **** p < 0.0001.

KC3AC1 cells were incubated for 6 h under an isotonic or hypertonic medium. Then, TLDA and miRNAs-Seq allowed us to establish a shortlist of five miRNA candidates, the expression of which was induced under hypertonicity with a fold-induction ≥1.5: miR-135a-5p, miR-28a-3p, miR-30c-2-3p, miR-324-5p, miR-335-5p. We could locate binding sites for these miRNAs in murine *Nr3c2* (MR) 3′-UTR using Targetscan/(http://www.targetscan.org/vert_72/, accessed on 19 March 2021) [45] and miRmap/(https://mirmap.ezlab.org/app/, accessed on 19 March 2021) [46] software. miR-335-5p and miR-324-5p present with one binding site in the *Nr3c2* (MR) 3′-UTR, while miR-135a-5p, miR-28a-3p, and miR-30c-2-3p have two distinct binding sites (Figure 1A). Of note, binding sites for miR-28a-3p are very close, while that of miR-30c-2-3p and miR-135a-5p are near the stop codon and the polyA tail. Identification of such binding sites suggested that the *Nr3c2* (MR) transcript might be targeted by these miRNAs. We confirmed the regulation of these miRNA candidates by analyzing their expression profile in KC3AC1 cells using RT-qPCR. Following TLDA screening, RT-qPCR analyses demonstrated that hypertonicity represses MR expression (Figure 1B), confirming our previous observations [16,17]. miR-324-5p expression was significantly increased under hypertonicity; however, the expression of miR-135a-5p and miR-335-5p were not changed. Likewise, following the miRNAs-Seq screening, RT-qPCR analyses also confirmed that hypertonicity decreased MR expression while increasing miR-28a-3p and miR-30c-2-3p expression (Figure 1C). Because induction of miR-28a-3p expression was very weak (~10%) under hypertonicity, we further focused on miR-30c-2-3p and miR-324-5p.

### 3.2. miR-324-5p Directly Targets Nr3c2 (MR) and Elavl1 (HuR) Transcripts

**Figure 2:**
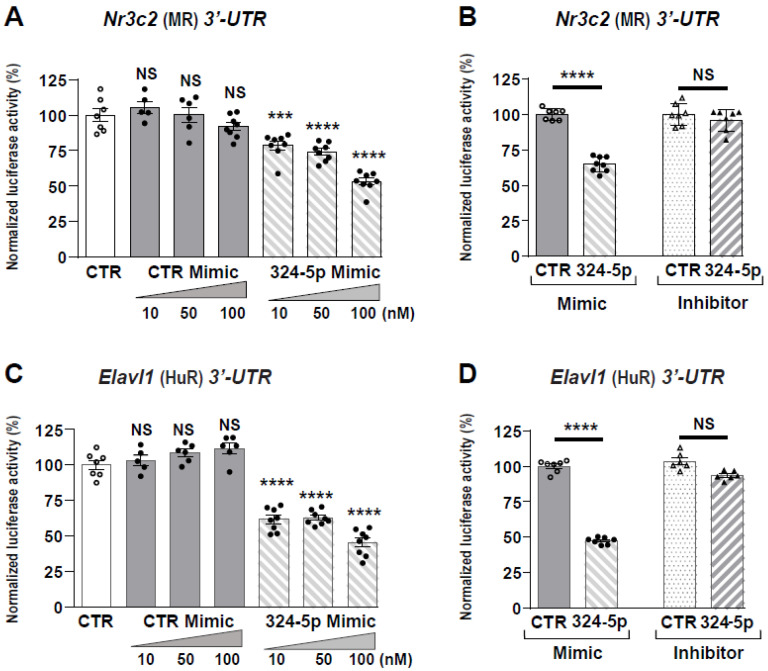
miR-324-5p functionally interacts with *Nr3c2* (MR) and *Elavl1* (HuR) 3′-UTR. The murine *Nr3c2* (MR) and *Elavl1* (HuR) 3′-UTR were cloned downstream of the pMIR-REPORT luciferase vector. (**A**,**C**) HEK 293T cells were transiently transfected, as described in Materials and Methods Section, with pMIR-luciferase plasmid (pMIR-Luc) fused to *Nr3c2* (MR) or *Elavl1* (HuR) 3′-UTR (40 ng/well of 96-well plates) and incubated with increasing concentrations (10, 50, 100 nM) of negative control Mimics (CTR Mimic) or of 324-5p Mimics. Luciferase activities were measured 24 h after transfection and normalized to β-galactosidase activities. (**B**,**D**) HEK 293T cells were transiently transfected with pMIR-Luc fused to *Nr3c2* (MR) or *Elavl1* (HuR) 3′-UTR (40 ng/well of 96-well plates) and 100 nM of CTR or 324-5p Mimics or with 100 nM of CTR or 324-5p Inhibitors. Luciferase activities were measured 24 h after transfection and normalized to β-galactosidase activities. Data are means ± SEMs (*n* = 8). NS = not significant, *** p < 0.001, **** p < 0.0001 compared to luciferase activity of pMIR-Luc fused to *Nr3c2* (MR) or *Elavl1* (HuR) 3′-UTR without Mimics (**A**,**C**) or with CTR Mimic or CTR inhibitor (**B**,**D**), arbitrarily set at 100%.

To demonstrate the functional interaction between miRNAs and *Nr3c2* (MR) transcript, we used the pMIR-Luc reporter plasmid fused to murine *Nr3c2* (MR) 3′-UTR [17]. HEK 293T cells were transfected with increasing concentrations (10 to 100 nM) of CTR or 324-5p Mimics. While CTR Mimics did not affect luciferase activity, 324-5p Mimics decreased luciferase activity in a dose-dependent manner, with a 40% maximum decrease reached with 100 nM Mimics (Figure 2A). This decrease in luciferase activity was blunted when cells were transfected with 100 nM 324-5p Inhibitors (Figure 2B), confirming that miR-324-5p directly targets the *Nr3c2* (MR) transcript. We also identified a binding site for miR-324-5p in *Elavl1* (HuR) 3′-UTR, located at +186. Therefore, we cloned the murine *Elavl1* (HuR) 3′-UTR downstream of luciferase cDNA in the pMIR-Luc plasmid. Transfection assays were next performed in HEK 293T with increasing concentrations (10 to 100 nM) of CTR or 324-5p Mimics, revealing that 324-5p Mimics also decreased luciferase activity by almost 40% with 10 nM 324-5p Mimics and by more than 60% with 100 nM 324-5p Mimics (Figure 2C). This decrease in luciferase activity was also blunted in the presence of 100 nM 324-5p Inhibitors (Figure 2D), confirming that miR-324-5p also directly targets the *Elavl1* (HuR) transcript [18,19].

### 3.3. miR-30c-2-3p Directly Targeted Nr3c2 (MR) and Elavl1 (HuR) Transcripts

**Figure 3:**
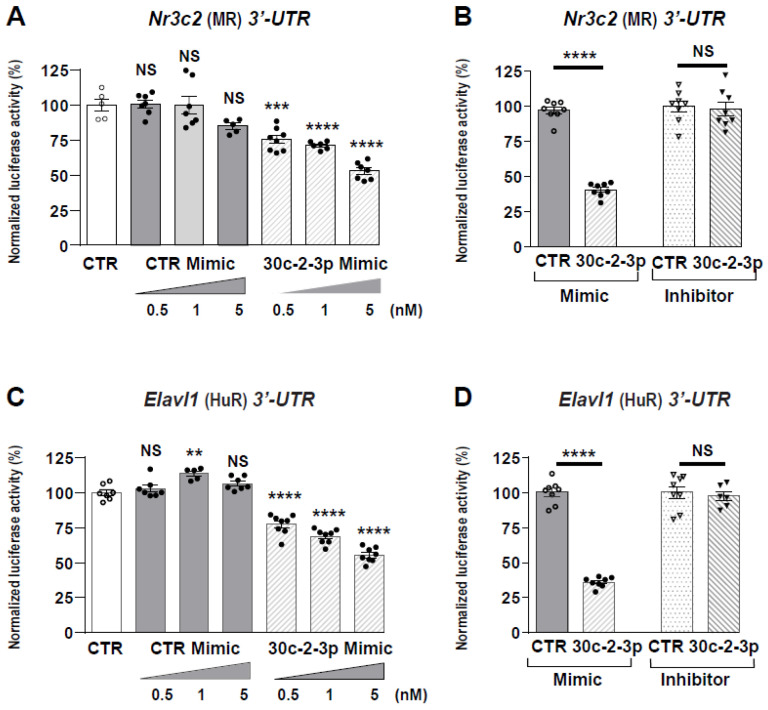
miR-30c-2-3p functionally interacts with *Nr3c2* (MR) and *Elavl1* (HuR) 3′-UTR. The murine *Nr3c2* (MR) and *Elavl1* (HuR) 3′-UTR were cloned downstream of the pMIR-REPORT luciferase vector. (**A**,**C**) HEK 293T cells were transiently transfected, as described in Materials and Methods Section, with pMIR-luciferase plasmid (pMIR-Luc) fused to *Nr3c2* (MR) or *Elavl1* (HuR) 3′-UTR (40 ng/well of 96-well plates) and incubated with increasing concentrations (0.5, 1, 5 nM) of negative control Mimics (CTR Mimic) or 30c-2-3p Mimics. Luciferase activities were measured 24 h after transfection and normalized to β-galactosidase activities. (**B**,**D**) HEK 293T cells were transiently transfected with pMIR-Luc fused to *Nr3c2* (MR) or *Elavl1* (HuR) 3′-UTR (40 ng/well of 96-well plates) and 10 nM of control (CTR) or 30c-2-3p Mimics and with 10 nM of control (CTR) or 30c-2-3p Inhibitors. Luciferase activities were measured 24 h after transfection and normalized to β-galactosidase activities. Data are means ± SEMs (*n* = 8). NS = not significant, ** p <0.01, *** p <0.001, **** p <0.0001 compared to luciferase activity of pMIR-Luc fused to *Nr3c2* (MR) or *Elavl1* (HuR) 3′-UTR without Mimics (**A**,**C**) or with CTR Mimics or CTR Inhibitors (**B**,**D**), arbitrarily set at 100%.

We used the same approach to show that miR-30c-2-3p directly targets *Nr3c2* (MR) and *Elavl1* (HuR) transcripts since we identified a binding site for miR-30c-2-3p in *Elavl1* (HuR) 3′-UTR. We transfected HEK 293T cells with increasing concentrations (0.5 to 5 nM) of CTR or 30c-2-3p Mimics. While CTR Mimics did not affect luciferase activity, 30c-2-3p Mimics significantly decreased luciferase activity in a dose-dependent manner, with a maximum of 50% decrease reached with 5 nM Mimics (Figure 3A), which was sufficient to obtain the maximum decrease in luciferase activity (Supplementary Figure S1). This effect was blunted when cells were transfected with 10 nM 30c-2-3p Inhibitors (Figure 3B), thus confirming that miR-30c-2-3p directly targets *Nr3c2* (MR) transcript. We next performed similar transfection assays and demonstrated that miR-30c-2-3p also directly targets *Elavl1* (HuR) transcript (Figure 3C,D). Altogether, Figure 2 and Figure 3 suggest that both miRNAs could alter MR signaling by destabilizing *Nr3c2* (MR) and/or *Elavl1* (HuR) transcripts.

### 3.4. Cooperativity between miRNAs and Tis11b Action

**Figure 4:**
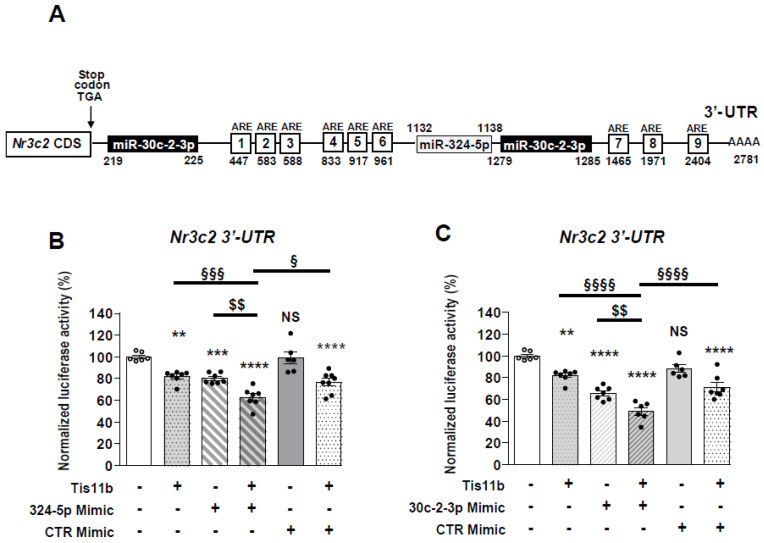
Cooperativity between miR-324-5p or miR-30c-2-3p and Tis11b on *Nr3c2* (MR) 3′-UTR. (**A**) Location of A-U rich response elements (ARE) motifs [18] and of putative binding sites for miR-324-5p and miR-30c-2-3p in mouse *Nr3c2* (MR) 3′-UTR, positioned after the stop codon TGA, arbitrarily set at +1. HEK 293T cells were transiently transfected, as described in Materials and Methods Section, with pMIR-luciferase plasmid (pMIR-Luc) fused to *Nr3c2* (MR) 3′-UTR (40 ng/well of 96-well plates) with or without a Tis11b-encoding plasmid (10 ng/well) and in the absence or presence of 10 nM Control Mimics (CTR mimic) or of 10 nM 324-5p Mimics (**B**) or 0.5 nM 30c-2-3p Mimics (**C**). Luciferase activities were measured 24 h after transfection and normalized to β-galactosidase activities. Data are means ± SEMs (*n* = 8). NS = not significant, ** p < 0.01, *** p < 0.001, **** p < 0.0001 compared to luciferase activity of pMIR-Luc fused to *Nr3c2* (MR) without Mimics, Tis11b or with CTR Mimics (CTR Mimic), arbitrarily set at 100%. ^§^ *p* < 0.05, ^§§^ *p* < 0.01, ^§§§^ *p* < 0.001 and ^§§§§^ *p* < 0.0001 between the two conditions indicated by line.

We previously demonstrated that Tis11b physically interacts with *Nr3c2* (MR) 3′-UTR by binding to AREs (Adenylate/uridylate-Rich Elements) under hypertonicity [17]. Figure 4A indicates the location of the nine AREs elements in murine *Nr3c2* (MR) 3′-UTR. The miR-324-5p binding site is positioned at the nucleotide +1132, between ARE6 and ARE7. miR-30c-2-3p presents with one binding site located near the stop codon, at nucleotide +219, and a second binding site located at nucleotide +1279. Therefore, we wondered whether Tis11b could cooperate with miRNAs because such cooperativity had previously been reported [47]. HEK 293T cells were transfected with the pMIR-Luc-*Nr3c2* (MR) 3′-UTR together with the Tis11b-encoding vector alone, with 324-5p or 30c-2-3p Mimics alone or in combination. Figure 4B indicates that 324-5p Mimics decreased luciferase activity by 20%, while 30c-2-3p Mimics decreased it by 30% (Figure 4C) even in the presence of low concentrations of Mimics (10 nM and 0.5 nM, respectively). As expected, a low amount of Tis11b (10 ng/well) also decreased luciferase activity by 20% (Figure 4B,C). Furthermore, luciferase activity decreased by 40% to 50% when HEK 293T cells were transfected simultaneously with Tis11b and either 324-5p (Figure 4B) or 30c-2-3p Mimics (Figure 4C), thus providing evidence for cooperative action between miRNAs and Tis11b in destabilizing *Nr3c2* (MR) transcript.

### 3.5. miR-30c-2-3p Overexpression Impacts MR Expression and Signaling

**Figure 5:**
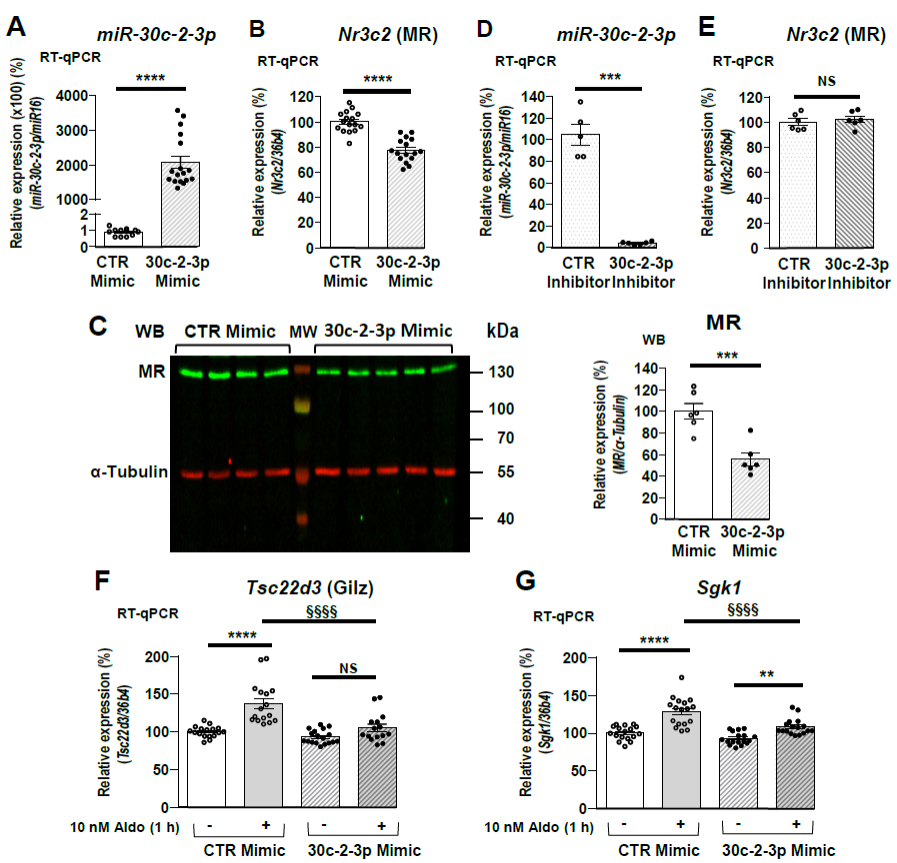
miR-30c-2-3p overexpression decreases MR expression in renal KC3AC1 cells and compromises MR-mediated target gene expression. Renal KC3AC1 cells were transfected with 10 nM Mimics or 10 nM negative Control Mimics (CTR mimic). (**A**) Specific overexpression of miR-30c-2-3p in renal KC3AC1 cells was confirmed by RT-qPCR. (**B**) Quantitative RT-qPCR of endogenous *Nr3c2* (MR) expression, analyzed 18 h after transfection. Data are means ± SEM from three independent experiments performed in 4–6 replicates (*n* = 12–18). NS = not significant, **** p < 0.0001 compared to CTR Mimics, arbitrarily set at 100%. (**C**) Western blot analysis of MR expression, 48 h following transfection of CTR Mimics or 30c-2-3p Mimics (left panel) and quantification of the corresponding signals (right panel) in which MR expression with CTR Mimics was arbitrarily set at 100%. Data are means ± SEMs (*n* = 6). (**D**,**E**) Renal KC3AC1 cells were transfected with 10 nM Inhibitors or negative Control Inhibitors (CTR inhibitor). (**D**) Specific overexpression of miR-30c-2-3p in renal KC3AC1 cells was confirmed by RT-qPCR. (**E**) Quantitative RT-qPCR of endogenous *Nr3c2* (MR) expression, analyzed 18 h after transfection. Data are means ± SEMs (*n* = 6). NS = not significant, *** p < 0.001, **** p < 0.0001 compared to CTR inhibitor, arbitrarily set at 100%. (**F**,**G**) Overexpression of miR-30c-2-3p prevented aldosterone-induced expression of *Tsc22d3* (Gilz) (**F**) or of Sgk1 (**G**) in renal KC3AC1 cells. KC3AC1 cells were deprived for 24 h in minimal medium then cells were transfected with 10 nM CTR Mimics or 10 nM 30c-2-3p Mimics. Eighteen hours later, cells were stimulated with 10 nM Aldosterone for 1 h then *Tsc22d3* (Gilz) or Sgk1 expression was measured by RT-qPCR. Data are means ± SEM from three independent experiments performed in six replicates (*n* = 18). NS = not significant, ** p < 0.01, **** p < 0.0001 compared to condition without aldosterone stimulation. *^§§§§^ p* < 0.0001 between the 2 conditions indicated by line.

RT-qPCR, performed on KC3AC1 cells transfected with 30c-2-3p Mimics, confirmed that miR-30c-2-3p expression was increased in transfected cells (Figure 5A), resulting in a 25% decrease in *Nr3c2* (MR) mRNAs (Figure 5B). As a result, MR protein (~130 kDa) levels were decreased by 50% in transfected cells compared to control (Figure 5C). We also transfected 30c-2-3p Inhibitors in KC3AC1 cells to demonstrate that miR-30c-2-3p specifically targets *Nr3c2* (MR) transcript (Figure 5D) and showed that complete inhibition of miR-30c-2-3p expression had no impact on MR expression (Figure 5E). Thereafter, KC3AC1 cells were cultivated for 24 h in a minimal medium and then stimulated for 1 h with 10 nM aldosterone following transfection of 10 nM CTR or 30c-2-3p Mimics.

As expected, aldosterone stimulated the expression of *Tsc22d3* (Gilz), a classical MR target gene, when cells were transfected with CTR Mimics. However, this effect was reduced when cells were transfected with 30c-2-3p Mimics (Figure 5F). Similar results were obtained for the kinase Sgk1. Aldosterone induced an increase in Sgk1 expression, but this stimulatory effect was weaker in the presence of 10 nM 30c-2-3p Mimics (Figure 5G). Taken together, our findings highlight a critical role for miR-30c-2-3p in compromising MR signaling following the reduction in MR expression in KC3AC1 cells.

### 3.6. Impairment of MR Signaling in KC3AC1 Cells Stably Expressing miR-324-5p

**Figure 6:**
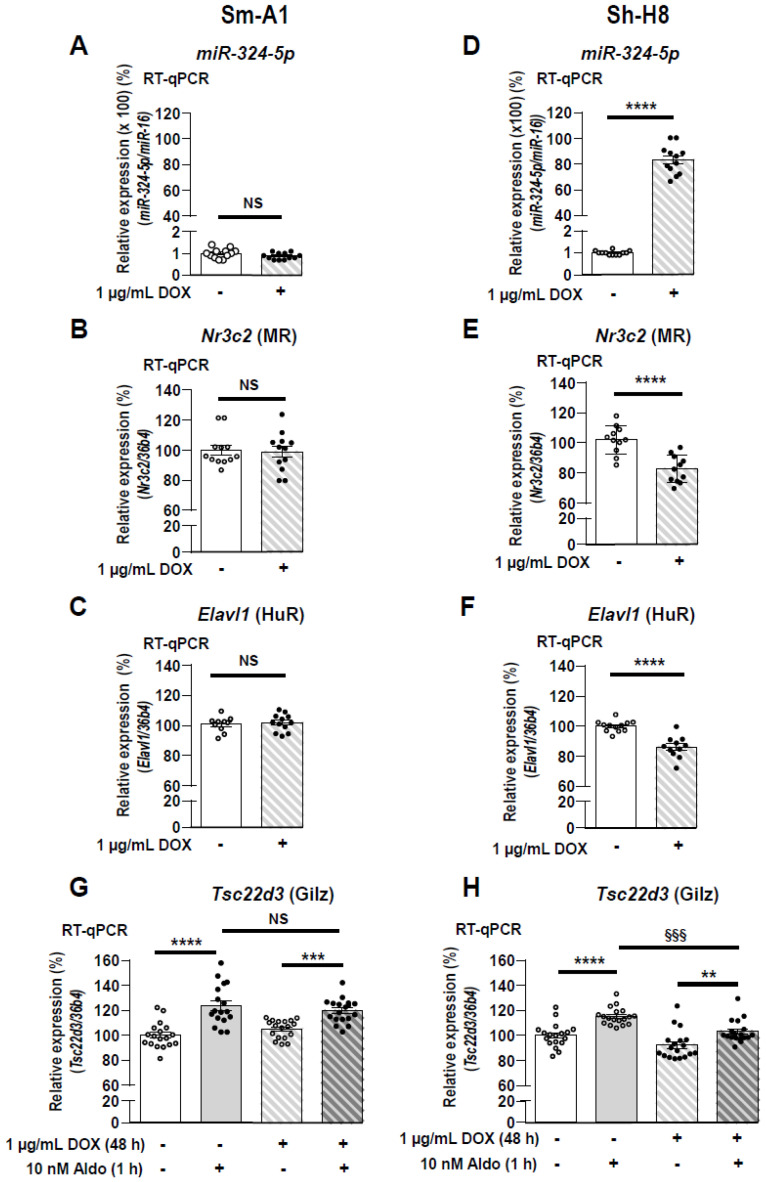
Impairment of MR signaling in KC3AC1 cells stably expressing miR-324-5p. miR-324-5p (**A**,**D**), *Nr3c2* (MR) (**B**,**E**) and *Elavl1* (HuR) (**C**,**F**) expression were determined by RT-qPCR, 48 h after doxycycline induction (1 µg/mL) in KC3AC1 clones stably transduced with lentiviral particles expressing inducible scrambled miRNAs (Sm-A1 clone, left panels) or miR-324-5p (Sh-H8 clone, right panels). Data are means ± SEMs from two independent experiments performed in six replicates (*n* = 12). NS = not significant, *** *p* < 0.001, **** *p* < 0.0001 compared to the condition in absence of doxycycline, arbitrarily set at 100%. (**G**,**H**) Sm-A1 and Sh-H8 clones were deprived in minimal medium for 48 h and incubated for 48 h with 1 µg/mL doxycycline. Thereafter, Sm-A1 and Sh-H8 clones were stimulated for 1 h with 10 nM aldosterone and *Tsc22d3* (Gilz) expression (**G**,**H**) was quantified by RT-qPCR where basal Gilz expression in renal cells, in the absence of DOX and aldosterone, was arbitrarily set at 100%. Data are means ± SEMs from three independent experiments performed in six replicates per condition (*n* = 18). NS = not significant. ** p < 0.01 *** p < 0.001 **** p < 0.0001 compared to the condition without aldosterone stimulation, arbitrarily set at 100%. ^§§§^ indicate *p* < 0.001 between the two conditions indicated by line.

Because transient transfection with high concentrations of 324-5p Mimics had no impact on MR expression, we established a stably transduced renal KC3AC1 cell line with lentivirus expressing inducible miR-324-5p to evaluate the impact of its overexpression on MR expression and signaling. KC3AC1 cells were transduced with scrambled or miR-324-5p ShMIMIC lentiviral particles. After puromycin selection (1.5 µg/mL) and doxycycline induction (1 µg/mL), cells were subjected to serial dilutions to finally establish two cellular clones: Sm-A1 clone expressing scrambled miRNAs (Figure 6, left panels) and Sh-H8 clone expressing miR-324-5p under doxycycline induction (Figure 6, right panels). Given that expression of miRNAs was coupled to that of the red fluorescent protein, we used fluorescence microscopy and RT-qPCR to select transduced clones that overexpressed either scrambled miRNAs or miR-324-5p (Supplementary Figure S2). Left panels in Figure 6A indicate that incubation of Sm-A1 clone for 48 h with doxycycline had, as expected, no impact on miR-324-5p expression. Therefore, *Nr3c2* (MR) and *Elavl1* (HuR) expressions were not impaired (Figure 6B,C). Conversely, miR-324-5p overexpression (×80) (Figure 6D) induced a significant 20 % decrease in *Nr3c2* (MR) and *Elavl1* (HuR) transcripts in Sh-H8 clone (Figure 6E,F). Finally, we determined the impact of miR-324-5p overexpression on MR expression and signaling: Sm-A1 clone and Sh-H8 clone were cultivated for 48 h in a minimal medium then stimulated for 1 h with 10 nM aldosterone. As expected, aldosterone induced a similar 20% increase in *Tsc22d3* (Gilz) expression when the Sm-A1 clone was incubated in the absence or presence of doxycycline (Figure 6G). However, this aldosterone-induced expression of *Tsc22d3* (Gilz) was reduced when the Sh-H8 clone was incubated in the presence of doxycycline (Figure 6H). Altogether, these findings demonstrate that miR-324-5p also impairs MR signaling in KC3AC1 renal cells.

### 3.7. Expression of miR-324-5p and miR-30c-2-3p Are Increased in Kidneys of Mice Treated with Furosemide

**Figure 7:**
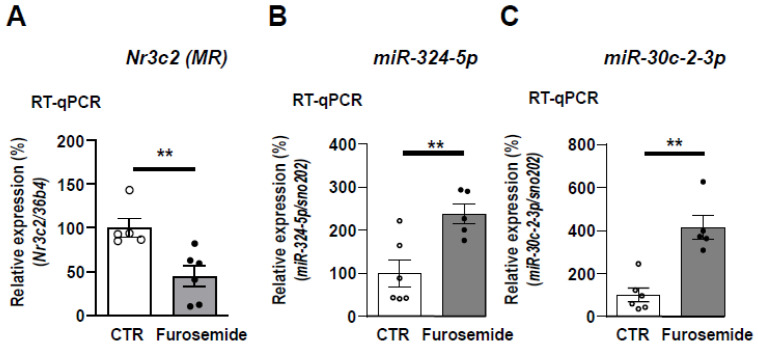
Treatment of mice with furosemide increases renal miR-324-5p and miR-30c-2-3p expression in vivo. Furosemide (40 mg/kg), an NKCC2 inhibitor, was administrated for 4 h before sacrifice to induce a relative luminal hypertonicity. RT-qPCR of *Nr3c2* (MR) (**A**), miR-324-5p (**B**), and miR-30c-2-3p (**C**) expression in the kidneys of treated-mice compared to control mice, arbitrarily set at 100%. Data are means ± SEMs (*n* = 5–6 animals). ** p < 0.01 compared to control mice.

To address the physiologic relevance of data obtained in KC3AC1 cells, we used kidney samples of mice treated for 4 h with furosemide, an NKCC2 inhibitor. In the cortex, basolateral fluid is maintained isotonic by vascularization, while luminal fluid, delivered at the end of the distal loop, is hypotonic due to the removal of salt in this segment impermeable to water. Thus, furosemide, by acutely inhibiting salt reabsorption in thick limbs, leads to an increase in the osmolality of fluid delivered to the distal nephron and to a decrease in MR expression (Figure 7A), as previously published [17]. Thus, we demonstrated that miR-324-5p and miR-30c-2-3p expression were dramatically increased in the kidneys of these furosemide-treated mice (Figure 7B,C), thus demonstrating, in vivo, that increased luminal tonicity, enhanced miR-324-5p and miR-30c-2-3p expressions. However, this increase in miRNAs expression in the whole kidney may be the result of changes not only in the mineralocorticoid sensitive distal nephron but also in other segments in the kidney, where MR and miRNAs may be co-expressed. We, therefore, established the quantitative expression profile of miRNAs (miR-324-5p and miR-30c-2-3p) along the nephron of three adult mice by performing glomeruli and nephron segments microdissection: the following structures were carefully microdissected according to morphologic and topographic criteria: glomeruli (Glo), proximal convoluted tubules (PCT), medullary and cortical thick ascending limb of Henle’s loop (mTAL and cTAL), connecting tubules (CNT), cortical and outer medullary collecting duct (CCD and OMCD) (Supplementary Figure S3A). This manual method of tubule selection allowed us to separate renal segments with a minimum of contamination, if any, of one type with another [48]. As shown in Supplementary Figure S3B below, relative MR expression is, as expected, weak in the glomeruli (Glo), the proximal tubule (PCT), and the Henle’s loop (mTAL and cTAL). By contrast, and as expected, MR expression is highest in the aldosterone-sensitive distal nephron (CNT, CCD, OMCD). This quantitative expression profile of miRNAs along the nephron, although difficult to implement, enabled us to confirm that miR-324-5p and miR-30c-2-3p are co-expressed in the aldosterone-sensitive distal nephron together with MR but they are also expressed in other segments such as Glo and PCT (Supplementary Figure S3C,D).

## 4. Discussion

**Figure 8:**
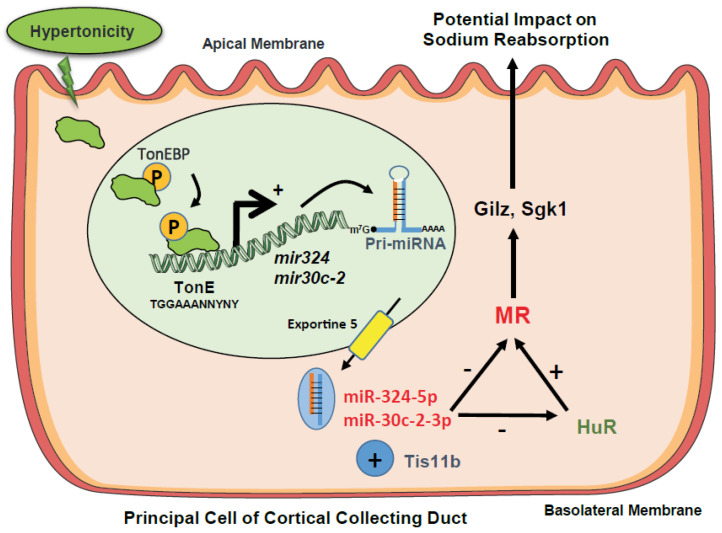
Proposed model for the post-transcriptional control of renal MR expression by miRNAs under hypertonicity and its impact on MR signaling. Under hypertonicity, the transcription factor TonEBP binds TonE elements located in the regulatory regions of *mir324* and *mir30c2* genes which encode miR-324-5p and miR-30c-2-3p, respectively. Hypertonicity stimulates transcription of pri-miRNAs of miR-324-5p and of miR-30c-2-3p and their maturation. Thereafter, these miRNAs can repress MR expression directly by interacting with *Nr3c2* (MR) 3′-UTR or indirectly by interacting with *Elavl1* (HuR), an RNA Binding Protein, which was shown to stabilize *Nr3c2* (MR) transcript under hypotonicity [19]. Moreover, these miRNAs can cooperate with Tis11b, an RNA Binding Protein, which was previously shown to accelerate destabilization of MR transcripts [18]. Thus, hypertonicity may compromise MR signaling.

We previously showed that MR expression is regulated by variations of extracellular tonicity prevailing in nephron segments [16] and highlighted the predominant role of RBPs [17,18,19]. Herein, we demonstrate that miRNAs also modulate MR expression in vitro and in vivo. Using high throughput screening approaches, we identified miR-324-5p and miR-30c-2-3p, expression of which is up-regulated under hypertonicity in renal cells and in kidneys of furosemide-treated mice, whereas MR expression is, in parallel, reduced. Renal cells resist hypertonicity by recruiting TonEBP (TonE Binding Protein), which belongs to the Rel factors family [49,50]. This transcription factor binds Tonicity response Elements (TonE), located in promoter regions of target genes such as *Aldose Reductase* [51] or *Zfp36l1* gene, which encodes Tis11b [17]. Jaspar software [52] identified TonE consensus sequence TGGAAANNYNY in regulatory regions of murine *mir324* and *mir30c2* gene (Supplementary Figure S4), suggesting that TonEBP may regulate the expression of these miRNAs (Figure 8). Moreover, we demonstrate that these miRNAs functionally interact with murine *Nr3c2* (MR) 3′-UTR, which is conserved among species and presents with the highest number of putative binding sites for miRNAs compared to other transcripts of the renin-angiotensin-aldosterone system [34]. Indeed, transfection of Mimics for both miRNAs decreased *Nr3c2* (MR) 3′-UTR-driven luciferase activity, an effect that was blunted by the corresponding Inhibitors. Given that cooperativity between RBPs and miRNAs had previously been reported [47], we transfected 324-5p or 30c-2-3p Mimics together with Tis11b in HEK 293T cells and demonstrated that they cooperate to decrease *Nr3c2* (MR) 3′-UTR-driven luciferase activity. This latter result could be explained by the location of AREs in *Nr3c2* (MR) 3′-UTR, on which Tis11b binds, located close to binding sites for miR-324-5p and miR-30c-2-3p, thus facilizing MR-destabilizing action of Tis11b [17]. Indeed, Grimson et al. demonstrated the functional importance of the environment surrounding miRNAs binding sites for their destabilizing action [53]. We also showed that miR-324-5p and miR-30c-2-3p could directly affect the stability of the *Nr3c2* transcript (MR) and/or indirectly destabilize the *Elavl1* transcript (HuR), a master RBP, which was shown to enhance MR expression and signaling under hypotonicity [18] (Figure 8). Such a complex interplay between these two classes of regulators is now coming to light [47]. Next, we analyzed the impact of miR-30c-2-3p and miR-324-5p overexpressions on MR expression in KC3AC1 cells. miR-30c-2-3p is more effective than miR-324-5p in destabilizing *Nr3c2* (MR) 3′-UTR since its overexpression, following transfection of low concentrations of Mimics, led to a significant decrease in MR expression, both at the messenger (25%) and protein (50%) levels, compromising MR signaling with blunted responses in terms of aldosterone-regulated gene expression (Figure 8). In contrast, overexpression of miR-324-5p did not affect MR expression even with high concentrations of Mimics. Consequently, the use of lentivirus expressing miR-324-5p has proven to be an efficient strategy to overexpress this miRNA in fully differentiated renal principal KC3AC1 cells, which fully express functional MR when high confluency is reached. Thus, we showed that the Sh-H8 clone presented with a 20 % decrease in MR expression at the mRNA level responsible for a reduced aldosterone-induced expression of *Tsc22d3* (Gilz), thus demonstrating that this miRNA also impairs MR signaling (Figure 8). This difference in efficacy could be explained by the number of binding sites for these miRNAs: there are two binding sites for miR-30c-2-3p at nucleotide +219 and +1279, whereas only one binding site for miR-324-5p is located at nucleotide +1131. Indeed, Doench et al. reported that additivity between several identical binding sites for a miRNA might have a higher impact on its repressive action [54]. Another possible explanation relies on the location of the miR-30c-2-3p binding sites, which are close to the stop codon, thus accelerating MR protein degradation [53]. Although the decrease in MR expression was relatively small (50% decrease in protein level), this action of miRNAs on the fine post-transcriptional control of MR expression is reminiscent of the role of miRNAs as buffers against variation in gene expression, as described by Ebert et al. [55]. Our study highlighted the role of these miRNAs, which could impair MR signaling in KC3AC1 cells. miR-30c-2-3p belongs to the miR-30 family involved in various renal diseases [56,57]. In addition, it has been reported that levels of miR-30 family members (including miR-30a, miR-30c, and miR-30e) increased in plasma of contrast-induced nephropathy (CIN) rats, suggesting that the miR-30 family might serve as early biomarkers for CIN [58]. Finally, miR-30c was also shown to be involved the in regulation of tubular cells apoptosis in cisplatin-induced nephrotoxicity [59]. Thus, our results add to the diversity of miR-30 family actions in renal pathophysiology. miR-324-5p is located on chromosome 11 and inhibits diverse functions in cancer. A recent study demonstrated that miR-324-5p plays a protective role against hepatocellular carcinoma [60]. Another study indicated significant upregulation of miR-324-5p in lung cancer cells and demonstrated that it promotes proliferation [61]. Although few data are available on its action in the kidney, we could not exclude the role of this miRNA in other MR target tissues. Indeed, miR-324-5p was demonstrated to contribute to seizure onset by repressing the expression of Kv4.2, a potassium channel, in the central nervous system [62], where MR plays a neuroprotective role [63,64]. Moreover, miR-324-5p was shown to inhibit mitochondrial fission, apoptosis, and myocardial infarction [65] through downregulation of Mtfr1, whereas MR is expressed in cardiac myocytes, and its activation is associated with myocardial hypertrophy and fibrosis [66,67]. To address the physiologic relevance of our study, we treated mice for 4 h with furosemide, a diuretic known to increase the relative luminal hypertonicity and decrease MR expression [17] and showed that miR-324-5p and miR-30c-2-3p expressions were increased in kidneys of these animals by 2- to 4-fold, respectively. This increase was higher than that observed in KC3AC1 cells, suggesting that their expression may also be increased in other nephron segments. Finally, our group has identified a restricted temporal window during renal development where MR signaling is ineffective due to specific downregulation of MR expression in the perinatal period compared to the lung, where MR expression is constant [68]. In this context, it would be interesting to evaluate whether such regulatory mechanisms are implicated in the regulation of MR expression within the perinatal period.

## 5. Conclusions

Altogether, our study has deciphered a novel regulatory mechanism involving the recruitment of miRNAs in the control of renal MR expression under hypertonicity, resulting in the alteration of MR signaling with a potential impact on sodium reabsorption. Our findings may also account for (patho)physiological situations affecting MR signaling in nephropathies [10], such as diabetic nephropathies or partial aldosterone resistance in newborns [8]. Given that miRNAs are also secreted in body fluids [69], these miRNAs may be quantified in plasma and urine of patients presenting with an altered salt and water balance or with diabetic nephropathy and might be used as non-invasive biomarkers to follow the evolution of renal diseases.

## Data Availability

Not applicable.

## Supplementary Materials

The following supporting information can be downloaded at: https://www.mdpi.com/article/10.3390/cells11091377/s1, Figure S1: miR-30c-2-3p functionally interacts with Nr3c2 (MR) 3’-UTR. Figure S2: Morphology of Sm-A1 and Sh-H8 clones; Figure S3: MR and miR-30c-2-3p, miR-324-5p expression in different nephron segments; Figure S4: Identification of half sites of Tonicity response Elements (TonEs) in regulatory sequences of miRNAs loci (red box).
