# Supplementary material for: miR-324-5p and miR-30c-2-3p Alter Renal Mineralocorticoid Receptor Signaling under Hypertonicity
Source: Cells. 2022 Apr 19;11(9):1377. doi: 10.3390/cells11091377 (PMC9104010; doi:10.3390/cells11091377)
Supplement: Supplementary file 1 [file cells-11-01377-s001.zip › cells-1642919-supplementary.pdf]

## Supplementary Tables

**Table S1** miRNA Stem-loop and TaqMan probes for miRNAs RT-qPCR

| miRNAs | Accession Number | Sequences | ID |
|---|---|---|---|
| mmu-miR-135a-5p | MIMAT0000147 | TATGGCTTTTTATTCCTATGTGA | 000460 |
| mmu-miR-28a-3p | MIMAT0004661 | CACTAGATTGTGAGCTGCTGGA | 002545 |
| mmu-miR-30c-2-3p | MIMAT0005438 | CTGGGAGAAGGCTGTTTACTCT | 002110 |
| mmu-miR-324-5p | MIMAT0000555 | CGCATCCCCTAGGGCATTGGTGT | 000539 |
| mmu-miR-335-5p | MIMAT0000766 | TCAAGAGCAATAACGAAAAATGT | 000546 |
| mmu-miR-16 | MIMAT0000527 | TAGCAGCACGTAAATATTGGCG | 000391 |
| snoRNA 202 | AF357327 | GCTGTACTGACTTGATGAAAGTACTTTTGAACCCTTTTCC ATCTGATG | 001232 |

**Table S2** Primer sequences

*All primer sequences are shown from 5' to 3'.*

**Table S2A** Primer sequences used for RNAs RT-qPCR

| Name | Accession Numbers | Amplicon size (pb) | Forward primer | Reverse primer |
|---|---|---|---|---|
| *Nr3c2* (MR) | M36074 | 153 | ATGGAAACCACACGGTGACCT | AGCCTCATCTCCACACACCAAG |
| *zfp36l1* (Tis11b) | NM_007564.3 | 100 | CGACACACCAGATCCTAGTCCTT | TGCATAAAACTTCGCTCAAGTCA |
| *Elavl1 (*HuR*)* | NM_010485.3 | 87 | CAGCCAATCCCAACCAGAA | TGGTGTACAGGGCCTCCAAA |
| *Tsc22d3 (*Gilz) | NM_010286.3 | 79 | CTGCTGTGGAGTTTGTGACATACTAG | CCAGGCAGGCACTTCTAAGCT |
| *Sgk1* | *AF205855* | *150* | TCACTTCTCATTCCAGACCGC | ATAGCCCAAGGCACTGGCTA |
| *18S* | X00686 | 66 | CCCTGCCCTTTGTACACACC | CGATCCGAGGGCCTCACTA |
| *Rplp0* (36b4) | NM_007475.5 | 128 | AGCGCGTCCTGGCATTGTCTGT | GGGCAGCAGTGGTGGCAGCAGG |

**Table S2B** Primer sequences used for cloning the pMIR-mHuR-3'-UTR

| Name | Forward primer | Reverse primer |
|---|---|---|
| pMIR-mHuR-3'UTR | ACGCACTAGTCGGAATAGATAATTAAGAGTGA | ACGCAGCTTCCACCTTTCTTTTTCTGA |

**Table S3** miRNAs Mimics & Inhibitors references for transfection

| Name | ID |
|---|---|
| 324-5p Mimic | MC10253 |
| 30c-2-3p Mimic | MC12646 |
| negative control Mimic | |
| 324-5p Inhibitor | MH10253 |
| 30c-2-3p Inhibitor | MH12646 |
| negative control Inhibitor | |

**Table S4** Antibodies used for Western blot analysis

| Name (Provider) | Species | Protein | Molecular weight (kDa) | Dilution |
|---|---|---|---|---|
| **Primary antibodies** | | | | |
| 39N | Rabbit | MR | 130 kDa | 1:1000 (cells)<br>1 :5000 (tissues) |
| α-Tubulin (Sigma) | Mouse | α-Tubulin | 50 kDa | 1:5000 |
| **Secondary antibodies** | | | | |
| Dylight Anti-Rabbit 800 (Fisher Scientific) | Goat | Rabbit IgG | | 1:10000 |
| Dylight Anti-Mouse 680 (Fisher Scientific) | Rabbit | Mouse IgG | | 1:10000 |

## Supplementary figure legends

**Supplementary Figure S1:** miR-30c-2-3p functionally interacts with *Nr3c2* (MR) 3'-UTR. HEK 293T cells were transiently transfected with pMIR-mMR-3'-UTR plasmid (40 ng/well of 96-well plates) and incubated with increasing concentrations (5 or 10 nM) of negative control Mimics (CTR Mimic) or 30c-2-3p Mimics. Luciferase activities were measured 24 h after transfection and normalized to β-galactosidase activities. *** indicated $P<0.001$. NS: non-significant.

**Supplementary Figure S2:** Morphology of Sm-A1 and Sh-H8 clones. Cells were grown for 5 days then were stimulated with 1 µg/mL Doxycyline for 48 h. **a, c** Cellular morphology of Sm-A1 clone (**a**) and Sh-H8 clone (**c**) observed with a phase-contrast microscope. Clones maintain a cuboid shape characteristics of epithelial cells and form domes at confluency suggesting a transepithelial ionic transport. **b, d** Sh-A1 (**b**) and Sh-H8 clones (**d**) were observed with a fluorescent microscope after 48 h of doxycycline stimulation. Fluorescence of turbo Red Fluorescent Protein allowed easy identification of transduced KC3AC1 cells, which should express either scrambled miRNAs (upper panels) or miR-324-5p (lower panels).

**Supplementary Figure S3:** MR and miR-30c-2-3p, miR-324-5p expression in different nephron segments. **a** Schematic representation of the different nephron segments; Proximal convoluted tubules (PCT), medullary and cortical thick ascending limb of Henle's loop (mTAL and cTAL), connecting tubules (CNT), cortical and outer medullary collecting duct (CCD and OMCD). **b, c, d** Quantification by RT-qPCR of MR (**b**), miR-30c-2-3p (**c**) and miR-324-5p (**d**) expression in different nephron segments of control mice.

**Supplementary Figure S4:** Identification of half sites of Tonicity response Elements (TonEs) in regulatory sequences of miRNAs loci (red box). **a** Schematic representation of *mir30c-2* gene on murine chromosome 1 (green bar) and location of TonEs, which were identified with Jaspar software in *mir30c-2* promoter region (blue bar). **b** Schematic representation of *mir324* gene on murine chromosome 11 (green bar) and location of TonEs, which were identified with Jaspar software in *mir324* promoter region (violet bar). The bent arrow represents the transcription start site (TSS) of *mir30c-2* (**a**) and *mir324* genes (**b**), encoding miR-30c-2-3p and miR-324-5p, respectively.

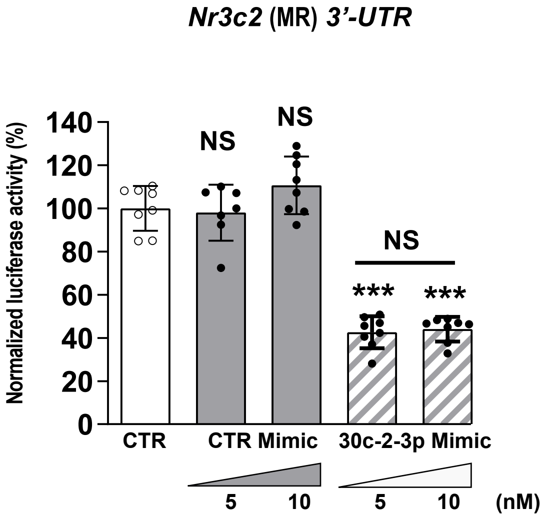


# Supplementary Figure S1

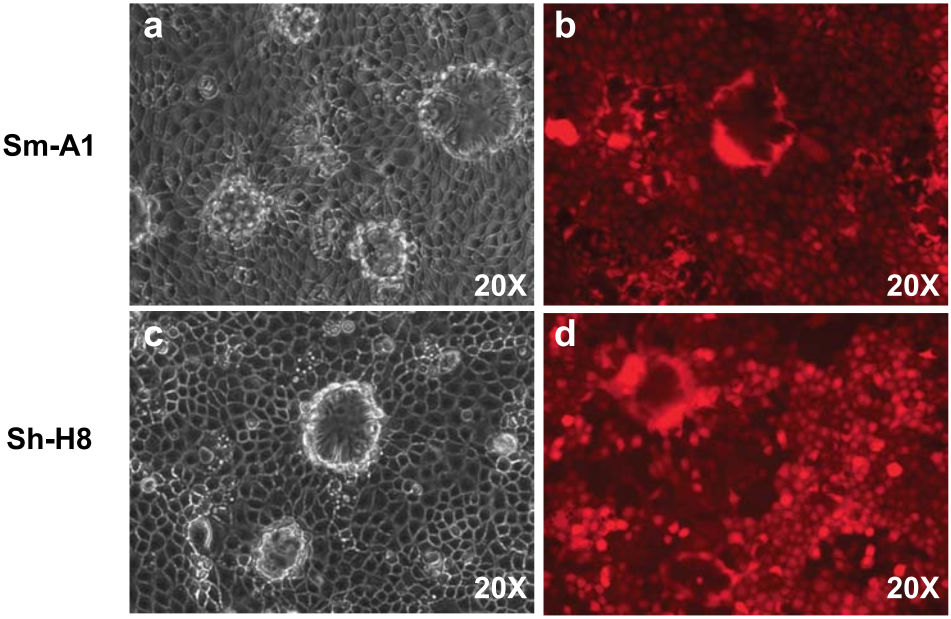


**Supplementary Figure S2**

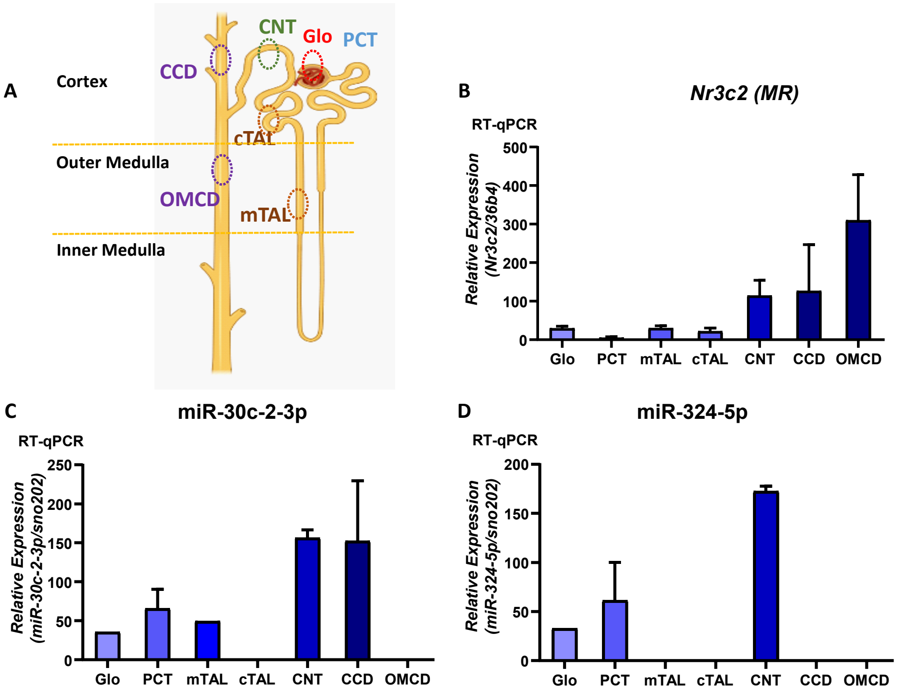


**Supplementary Figure S3**

A

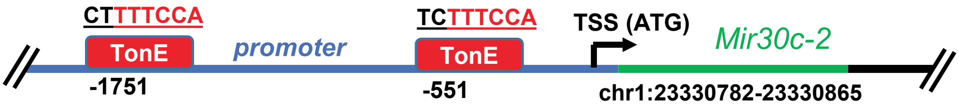


B

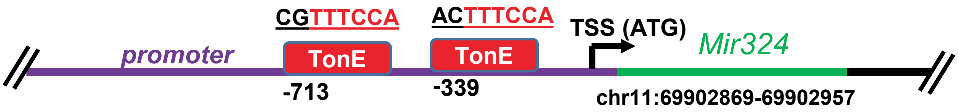


TonE : **TGGAAANNYNY**

**Supplementary Figure S4**
